# Uniportal video-assisted thoracic surgery: segmentectomy versus lobectomy—early outcomes

**DOI:** 10.1093/ejcts/ezae127

**Published:** 2024-03-28

**Authors:** Erik Sachs, Veronica Jackson, Mamdoh Al-Ameri, Ulrik Sartipy

**Affiliations:** Department of Molecular Medicine and Surgery, Karolinska Institutet, Stockholm, Sweden; Department of Cardiothoracic Surgery, Karolinska University Hospital, Stockholm, Sweden; Department of Molecular Medicine and Surgery, Karolinska Institutet, Stockholm, Sweden; Department of Molecular Medicine and Surgery, Karolinska Institutet, Stockholm, Sweden; Department of Cardiothoracic Surgery, Karolinska University Hospital, Stockholm, Sweden; Department of Molecular Medicine and Surgery, Karolinska Institutet, Stockholm, Sweden; Department of Cardiothoracic Surgery, Karolinska University Hospital, Stockholm, Sweden

**Keywords:** Pulmonary resections, Segmentectomy, Video-assisted thoracic surgery, Uniportal, Lung cancer

## Abstract

**OBJECTIVES:**

To assess the feasibility and safety of uniportal video-assisted thoracoscopic pulmonary segmentectomy compared with lobectomy by studying early postoperative outcomes.

**METHODS:**

We included all patients who underwent uniportal segmentectomy and lobectomy between 2017 and 2022 at Karolinska University Hospital. Early clinical outcomes were compared between the uniportal segmentectomy and lobectomy groups. Differences in baseline characteristics were addressed using inverse probability of treatment weighting.

**RESULTS:**

A total of 833 patients (232 segmentectomy, 601 lobectomy) were included. The number of uniportal operations increased during the study period. Patients in the segmentectomy and lobectomy groups, respectively, had stage I lung cancer in 65% and 43% of the cases; 97% and 94% had no postoperative complications, the median number of lymph node stations sampled was 4 vs 5, and non-radical microscopic resection occurred in 1.7% vs 1.8%. The drains were removed on postoperative day 1 in 75% vs 72% of the patients following segmentectomy and lobectomy, respectively, and 90% vs 89% were discharged directly home.

**CONCLUSIONS:**

Uniportal video-assisted segmentectomy was performed with similar early postoperative clinical results compared with uniportal lobectomy in patients with benign, metastatic or early-stage lung cancer.

**Figure:**
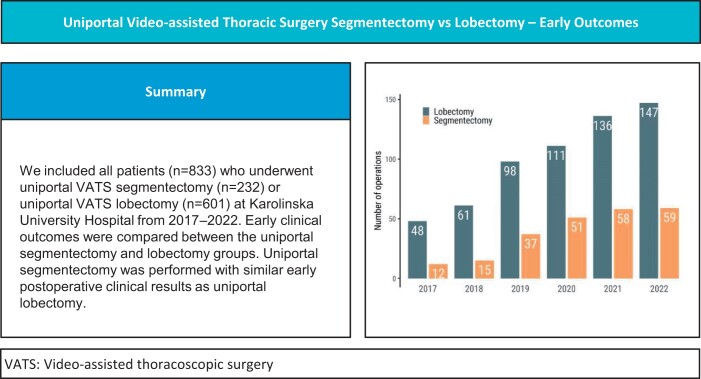

## INTRODUCTION

Anatomical resection by segmentectomy has recently become an accepted alternative to lobectomy in curative surgery for early-stage non-small-cell lung cancer [1]. Compared with lobectomy, segmentectomy entails a more complex surgical procedure owing to the challenging dissection of the bronchovascular structures. However, a meta-analysis comparing video-assisted thoracic surgery (VATS) segmentectomy and VATS lobectomy for stage I non-small-cell lung cancer showed that the 2 procedures were comparably safe [2]. For many years, sublobar resection for malignant disease has been considered an option only when lobectomy is contraindicated. However, recently, 2 important studies have given reason to consider sublobar resection with segmentectomy as an alternative to lobectomy in select cases of early-stage malignant disease [3, 4]. With increasing access to computed tomography and, thus, increased detection of early-stage lung cancer, minimally invasive surgical procedures that spare lung parenchyma are a desirable option. Additionally, guidelines regarding this topic are likely to be updated in the near future [5].

Uniportal VATS (uVATS) is a minimally invasive method of gaining surgical access to the pleural cavity. Surgery is performed through a single 3–4-cm opening without the use of rib spreading [6, 7]. uVATS segmentectomy has been demonstrated to be safe and oncologically effective in specialized, high-volume uniportal centres. [8–11]. uVATS lobectomy was successfully implemented at our centre in 2016 [12, 13]. However, until 2017, more technically complex procedures, such as segmentectomies, had rarely been performed through the uniportal approach. The aim of this study was to describe the feasibility and safety of introducing uVATS segmentectomies in our department, where uVATS lobectomies were routinely performed.

## PATIENTS AND METHODS

The need for patient informed consent was waived and ethical approval was granted by the Swedish Ethical Review Authority (Dnr: 2019-00964 approved 1 March 2019, 2021-00248 approved 16 February 2021 and 2022-07334-02 approved 9 April 2023).

### Study design

This was a retrospective, single-centre observational cohort study. Karolinska University Hospital is the only provider of thoracic surgery in Stockholm County and serves ∼2.5 million persons (25% of the total Swedish population). The REporting of studies Conducted using Observational Routinely-collected health Data (RECORD) statement and the Strengthening the Reporting of Observational Studies in Epidemiology (STROBE) statement guidelines for observational studies using routinely collected data were followed [14, 15].

### Study population and outcomes

Patients were identified through the Swedish national quality register for general thoracic surgery (ThoR, http://www.ucr.uu.se/thor). VATS was the standard approach, but the choice to plan for multiportal VATS (mVATS) or uVATS was at the operating surgeon’s discretion. All patients who underwent uVATS segmentectomy or lobectomy between 1 January 2017 and 31 December 2022 were included. An ‘as treated’ approach to analysis was used. Cases that were planned for uVATS resections but were converted to mVATS or thoracotomy were excluded. The main outcomes were early postoperative complications including all-cause mortality, thoracic drainage time, hospital length of stay and discharge home or to another clinic, as available from the ThoR registry. Information on vital status was obtained from the Swedish Population Register, and the Swedish Personal Identity Number made it possible to cross-link data at the individual level [16, 17].

### Definitions

Comorbidity was defined as a significant current medical condition possibly affecting prognosis or requiring treatment. Smoking status was categorized as current, former or never-smoker. Current smoker was defined as active smoker or smoking cessation within 1 month of surgery. Former smoker was defined as smoking cessation more than 1 month before surgery, and never-smoker was defined as never having smoked actively. Conversion to mVATS or open thoracotomy was defined as any instance where uVATS was intended but 1 or more thoracoscopic ports was added or when a thoracotomy was performed, regardless if under controlled or emergency circumstances. Definitions of complications are provided in Supplementary Material, Table S2.

### Surgical technique

The uniportal approach was as described by the consensus report from the uVATS Interest Group [18]. Briefly, surgery was performed through a single 4-cm incision in the mid-axillary line in the 4th or 5th intercostal space. A 10-mm, rigid, 30° high-definition camera (ENDOEYE HD II; Olympus, Hamburg, Germany) was inserted and held in place posteriorly in the port using a rubber ligature. Vessels, bronchi and intersegmental planes were divided using articulated endostaplers (ECHELON+; Ethicon Inc., Cincinnati, OH, USA and Endo GIA Ultra; Covidien Medtronic, Minneapolis, MN, USA). Dissection was performed using an ultrasonic thermal energy device (HARMONIC; Ethicon, Cincinnati, OH, USA) or an electrocautery blade hook. Local anaesthesia was initiated and administered continuously through a subpleural catheter placed along the same intercostal space as the incision, and single subpleural injections were administered in the intercostal spaces above and below. A 24-French drain was placed anteriorly or posteriorly in the incision and connected to a digital chest drainage and monitoring system (THOPAZ+; Medela Healthcare, Baar, Switzerland) at the end of every operation. Three-dimensional reconstruction was not routinely used in planning of surgery. In segmentectomies, bronchoscopy was used to confirm the division of the correct segmental bronchus and subsequent selective reinflation was used to delineate the segment that was to be resected. Frozen section of sampled lymph node stations and resection margins following segmentectomy was not performed. Intraoperative analysis of radicality or metastatic status was not performed on a routine basis, although we recognize that this is recommended when performing segmentectomy [5].

### Statistical methods

Baseline characteristics were presented as means and standard deviations for continuous variables. Categorical variables were presented as frequencies and percentages. We did not assess the distribution of continuous variables for normality. Inverse probability of treatment weighting was used to address baseline differences. Stabilized weights were derived from propensity scores generated using logistic regression models [19]. All variables reported in Table 1 were used in the estimation of the propensity scores. We examined the distribution of weights and found no observations with extreme weights, indicating that trimming was unnecessary. Balance between the lobectomy and segmentectomy groups was assessed by standardized mean differences. An absolute standardized difference of ≤0.1 was considered an ideal balance, and ≤0.2 is generally considered an acceptable balance [20]. We used Stata ‘svy’ Pearson’s chi-squared test to compare the weighted segmentectomy and lobectomy groups. Data management and statistical analyses were performed with R version 4.3.1 (R Foundation for Statistical Computing, Vienna, Austria) and Stata version 18.0 (StataCorp LLC, College Station, TX, USA) and included the use of the R packages WeightIt and cobalt [19, 21].

**Table 1:** Baseline characteristics in patients who underwent uniportal lobectomy or segmentectomy before and after inverse probability of treatment weighting

		Before weighting			After weighting		
Variable	Total population	Lobectomy	Segmentectomy	SMD	Lobectomy^a^	Segmentectomy^a^	SMD
Number of patients	833	601	232		605.4	229.5	
Age (years), mean (SD)	68.8 (10.4)	69.5 (10.0)	67.2 (11.0)	0.210	68.4 (11.2)	68.4 (9.91)	0.004
Sex, female	482 (57.9)	349 (58.1)	133 (57.3)	0.015	351.8 (58.1)	132.1 (57.5)	0.012
Body mass index (kg/m^2^), mean (SD)	25.9 (4.80)	25.8 (4.52)	26.2 (5.46)	0.078	25.8 (4.55)	25.7 (4.87)	0.014
Performance status >0	102 (12.2)	83 (13.8)	19 (8.2)	0.180	72.8 (12.0)	25.5 (11.1)	0.030
Smoking				0.199			0.018
Never	224 (26.9)	147 (24.5)	77 (33.2)		167.5 (27.7)	61.8 (26.9)	
Former	449 (53.9)	332 (55.2)	117 (50.4)		322.8 (53.3)	124.3 (54.2)	
Current	160 (19.2)	122 (20.3)	38 (16.4)		115.1 (19.0)	43.4 (18.9)	
Preoperative FEV1 (l), mean (SD)	2.43 (0.73)	2.43 (0.73)	2.45 (0.74)	0.028	2.44 (0.71)	2.46 (0.73)	0.029
No comorbidity	364 (43.7)	267 (44.4)	97 (41.8)	0.053	266.4 (44.0)	96.2 (41.9)	0.042
Ischaemic heart disease	85 (10.2)	62 (10.3)	23 (9.9)	0.013	63.3 (10.5)	24.9 (10.8)	0.013
Hypertension	389 (46.7)	270 (44.9)	119 (51.3)	0.128	281.2 (46.5)	110.6 (48.2)	0.034
Arrhythmia	83 (10.0)	61 (10.1)	22 (9.5)	0.022	59.9 (9.9)	24.4 (10.6)	0.024
Prior thoracic surgery	27 (3.2)	11 (1.8)	16 (6.9)	0.250	23.6 (3.9)	7.8 (3.4)	0.027
Heart failure	28 (3.4)	18 (3.0)	10 (4.3)	0.070	19.1 (3.2)	6.7 (2.9)	0.012
Diabetes	118 (14.2)	80 (13.3)	38 (16.4)	0.086	84.5 (14.0)	32.4 (14.1)	0.004
Prior stroke/TIA	51 (6.1)	40 (6.7)	11 (4.7)	0.083	35.6 (5.9)	11.9 (5.2)	0.031
Chronic kidney disease	27 (3.2)	18 (3.0)	9 (3.9)	0.049	18.4 (3.0)	6.5 (2.8)	0.012
Preoperative radiotherapy	12 (1.4)	10 (1.7)	2 (0.9)	0.072	8.3 (1.4)	2.2 (0.9)	0.039
Preoperative chemotherapy	31 (3.7)	23 (3.8)	8 (3.4)	0.020	22.8 (3.8)	8.0 (3.5)	0.015
Preoperative PET	773 (92.8)	571 (95.0)	202 (87.1)	0.281	555.3 (91.7)	211.4 (92.1)	0.014
Year of surgery				0.194			0.025
2017	60 (7.2)	48 (8.0)	12 (5.2)		44.0 (7.3)	17.7 (7.7)	
2018	76 (9.1)	61 (10.1)	15 (6.5)		54.4 (9.0)	21.2 (9.2)	
2019	135 (16.2)	98 (16.3)	37 (15.9)		97.5 (16.1)	37.3 (16.3)	
2020	162 (19.4)	111 (18.5)	51 (22.0)		116.5 (19.2)	44.5 (19.4)	
2021	194 (23.3)	136 (22.6)	58 (25.0)		144.8 (23.9)	54.5 (23.7)	
2022	206 (24.7)	147 (24.5)	59 (25.4)		148.1 (24.5)	54.3 (23.7)	

Numbers are *n* (%) unless otherwise noted.

a The overall numbers of patients in each group are not necessarily integers owing to inverse probability of treatment weighting.

FEV1: forced expiratory volume in 1 s; SD: standard deviation; SMD: standardized mean differences; PET: positron emission tomography; TIA: transient ischaemic attack.

### Missing data

The following variables had missing data: preoperative forced expiratory volume (4.6%) and body mass index (0.4%). Missing data were handled by imputing the median value in each group.

## RESULTS

**Figure 1:**
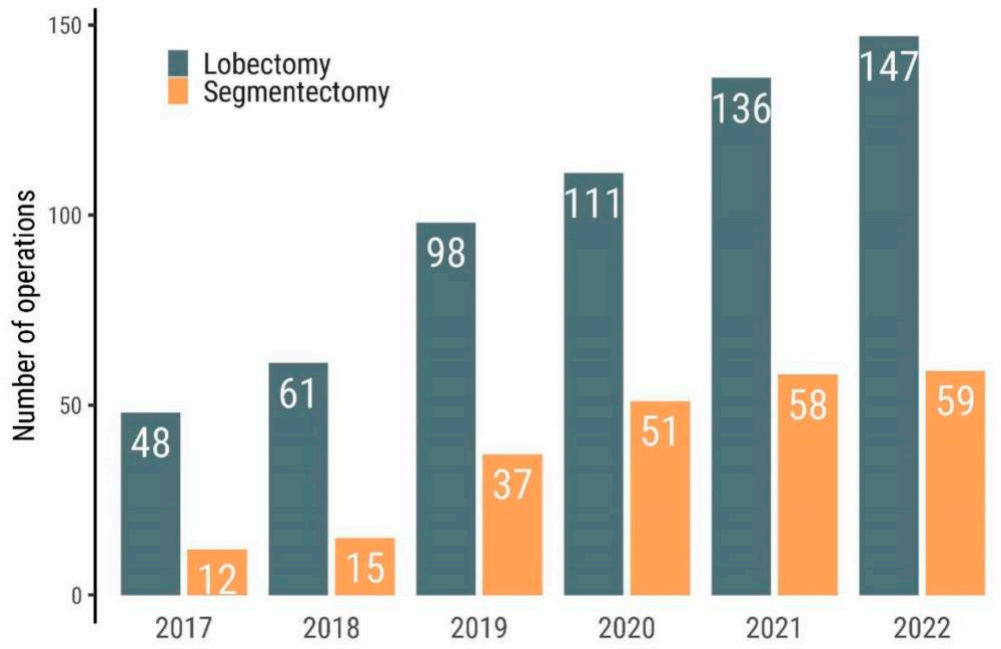
Number of uniportal VATS procedures per year. VATS: video-assisted thoracic surgery.

Between 2017 and 2022, uVATS lobectomy or segmentectomy was planned in 914 patients. Eighty-one cases were converted to mVATS or open thoracotomy and these patients were excluded from the final analysis (Supplementary Material, Fig. S1). Eight hundred thirty-three patients underwent uniportal anatomical lung resection comprising 601 lobectomies (72.2%) and 232 segmentectomies (27.8%) (Table 1). In total, there were 37 and 44 conversions to mVATS or open thoracotomy, respectively. In the segmentectomy group, there were 2 and 4 conversions to mVATS and thoracotomy, respectively, owing to technical difficulties. Details on conversions are presented in Table 2. The number and proportion of segmentectomies increased over the study period (Fig. 1); the resected segments are reported in Supplementary Material, Table S1. The mean age was higher for patients who underwent lobectomy versus segmentectomy at 69.5 years and 67.2 years, respectively (*P* = 0.006). Women comprised 58% of the patients in the lobectomy group and 57% in the segmentectomy group. Patients with a high performance status score (>0) were more numerous in the lobectomy group versus the segmentectomy group at 13.8% vs 8.2%, respectively (*P* = 0.36). Fewer patients who underwent lobectomy versus segmentectomy had undergone prior thoracic surgery, 1.8% vs 6.9% (*P* < 0.001). Preoperative positron emission tomography was performed more often in patients who underwent lobectomy versus segmentectomy at 95.0% vs 87.1%, respectively (*P* < 0.001). After weighting, the study groups were well-balanced for all baseline characteristics (Table 1 and Supplementary Material, Fig. S2).

**Table 2:** Conversions to mVATS or open thoracotomy

Year	mVATS 37 (46)	Thoracotomy 44 (54)	Total 81 (100)	Technical difficulties 50 (62)	Bleeding 19 (23)	Adhesions 8 (10)	Other 3 (4)
2017	2 (5)	6 (14)	8 (10)	7 (14)	1 (5)	0 (0)	0 (0)
2018	5 (14)	6 (14)	11 (14)	5 (10)	4 (21)	1 (13)	1 (33)
2019	8 (22)	12 (27)	20 (25)	11 (22)	4 (21)	3 (38)	2 (67)
2020	5 (14)	4 (9)	9 (11)	6 (12)	3 (16)	0 (0)	0 (0)
2021	16 (43)	8 (18)	24 (30)	19 (37)	2 (11)	3 (38)	0 (0)
2022	1 (3)	8 (18)	9 (11)	3 (6)	5 (26)	1 (13)	0 (0)

Numbers are *n* (%).

mVATS: multiportal video-assisted thoracoscopic surgery.

### Tumour stage and histologic subtypes

Stage 1A disease was identified in 49% of the patients (Table 3). Stage 1A disease was more common in the segmentectomy group versus the lobectomy group at 65% vs 43%, respectively. Adenocarcinoma was the most prevalent histologic subtype, accounting for 75% in the lobectomy group and 66% in the segmentectomy group. Patients with other malignancy than primary lung cancer, metastases or benign disease were more common in the segmentectomy versus lobectomy groups at 21% vs 6%, respectively. For benign disease only, the percentages were 7% for segmentectomy and 2% for lobectomy.

**Table 3:** Postoperative histopathology and pathologic stage

	Overall (*n* = 833)	Lobectomy (*n* = 601)	Segmentectomy (*n* = 232)	*P*-value
Stage				<0.001
IA	411 (49)	260 (43)	151 (65)	
IB	115 (14)	102 (17)	13 (6)	
IIA	112 (13)	101 (17)	11 (5)	
IIB	45 (5)	42 (7)	3 (1)	
IIIA–IV	58 (7)	54 (9)	4 (2)	
Other^a^	92 (11)	42 (7)	50 (22)	
Histopathology				<0.001
Adenocarcinoma	602 (72)	449 (75)	153 (66)	
Squamous cell carcinoma	70 (8)	60 (10)	10 (4)	
Carcinoid	46 (6)	32 (5)	14 (6)	
Other malignancy	30 (4)	24 (4)	6 (3)	
Metastasis	56 (7)	24 (4)	32 (14)	
Benign	29 (3)	12 (2)	17 (7)	

Numbers are *n* (%).

a Other: metastasis, benign or malignant (other).

### Complications

Four patients died during follow-up (Table 4). One patient died within 30 days and 3 within 90 days in the lobectomy group compared with 1 patient who died within 90 days in the segmentectomy group. There were no significant differences in perioperative mortality between the groups (30 days: *P* = 0.538; 90 days: *P* = 0.271).

**Table 4:** Postoperative events and complications after uniportal lobectomy or segmentectomy in the weighted population

Outcome	Lobectomy	Segmentectomy	*P*-value
Complications			
No complication	566 (94)	226 (97)	0.101
Pneumothorax requiring a new drain	11 (2.0)	2 (0.66)	0.138
Arrhythmia	2 (0.3)	3 (1.8)	0.040
Stroke/TIA	1 (0.2)	0 (0)	0.538
Myocardial infarction	0 (0)	0 (0)	−
Wound infection	0 (0)	0 (0)	−
Pneumonia	6 (0.1)	0 (0)	0.133
Empyema	1 (0.2)	0 (0)	0.538
Lymph leak	2 (0.3)	0 (0)	0.387
Recurrent nerve paralysis	1 (0.2)	0 (0)	0.538
Phrenic nerve paralysis	0 (0)	0 (0)	−
Pulmonary embolism	4 (0.7)	0 (0)	0.219
Other complication	7 (1.2)	1 (0.4)	0.346
Reoperation	21 (3.2)	6 (3.1)	0.933
Transfusion of blood products	8 (1.4)	7 (4.0)	0.034
Station 7 sampled^a^	500 (89)	130 (73)	<0.001
≥3 N2-stations sampled, including station 7^a^	396 (71)	94 (50)	<0.001
Incomplete resection (on microscopy)^b^	10 (1.8)	3 (1.7)	0.946
Death within 30 days	1 (0.2)	0 (0)	0.538
Death within 90 days	3 (0.6)	1 (0.2)	0.271

Numbers are *n* (%).

a Patients with benign disease or metastases were excluded.

b Patients with benign histopathology were excluded.

TIA: transient ischaemic attack.

Complications were rare in both groups; 94% and 97% of the patients had no complications following lobectomy and segmentectomy, respectively. The most common complication was reoperation for any reason at 3.2% and 3.1% for lobectomies and segmentectomies, respectively. The proportion of patients with pneumothorax who required a new drain did not vary significantly between the groups at 2.0% vs 0.66% for lobectomies versus segmentectomies, respectively (*P* = 0.138). Postoperative arrhythmia occurred in 0.3% of the patients in the lobectomy group and 1.8% in the segmentectomy group (*P* = 0.040). Transfusion of blood products was more common after segmentectomy versus lobectomy at 4.0% vs 1.4%, respectively (*P* = 0.034).

### Oncological outcomes

**Figure 2:**
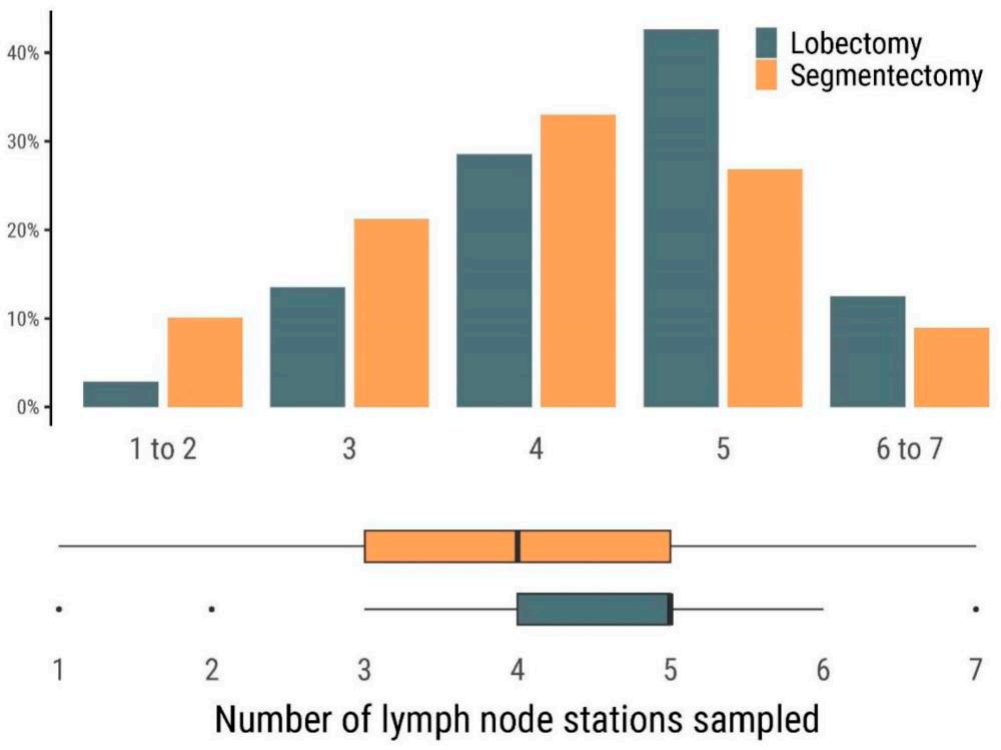
The upper panel shows the number of lymph node stations that were sampled in patients who underwent uniportal lobectomy or segmentectomy, and the lower panel shows a box plot of the number of lymph node stations that were sampled. The analysis was performed on the unweighted population and excluded patients if sampling was not performed (patients with benign disease or metastases).

Figure 2 shows the number of sampled lymph node stations in patients with primary malignancy. The extent of lymph node sampling differed significantly between the groups. Station 7 was sampled in 89% of lobectomies and 73% of segmentectomies (*P* < 0.001). Sampling of 3 or more N2-stations, including station 7, was performed in 71% of lobectomies and 50% of segmentectomies (*P* < 0.001). There was no difference in the rate of incomplete microscopic resection between the groups (*P* = 0.946) (Table 4).

### Length of stay and drainage time

**Figure 3:**
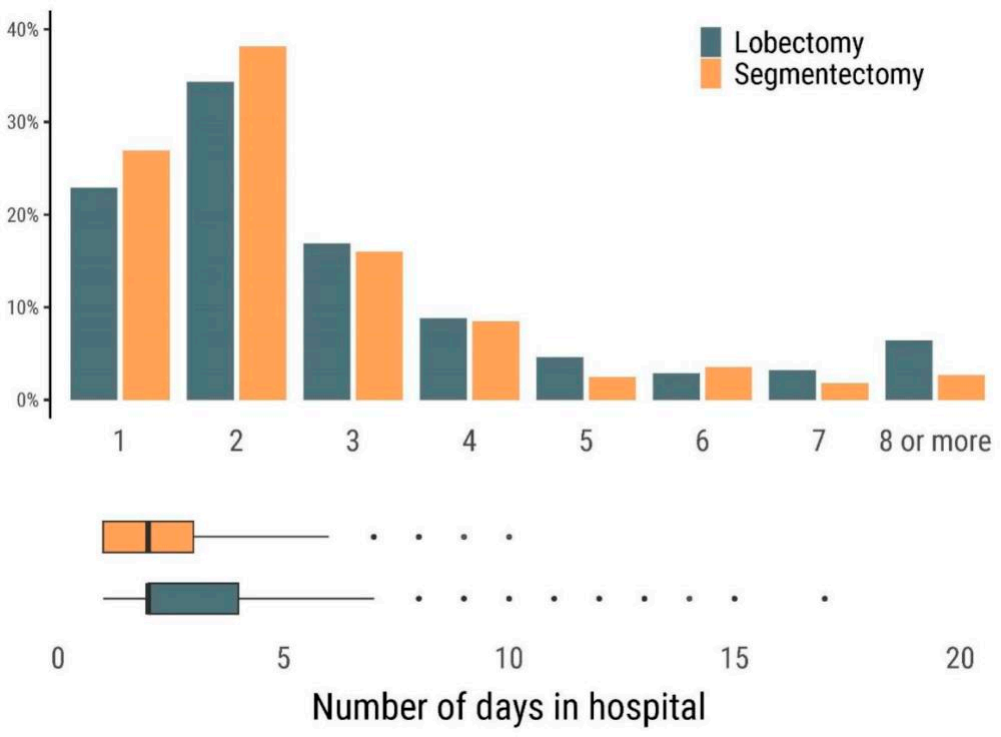
The proportion of patients according to days spent in hospital after uniportal lobectomy or segmentectomy. The lower panel shows a box plot of the length of stay. The analysis was performed on the weighted population.

**Figure 4:**
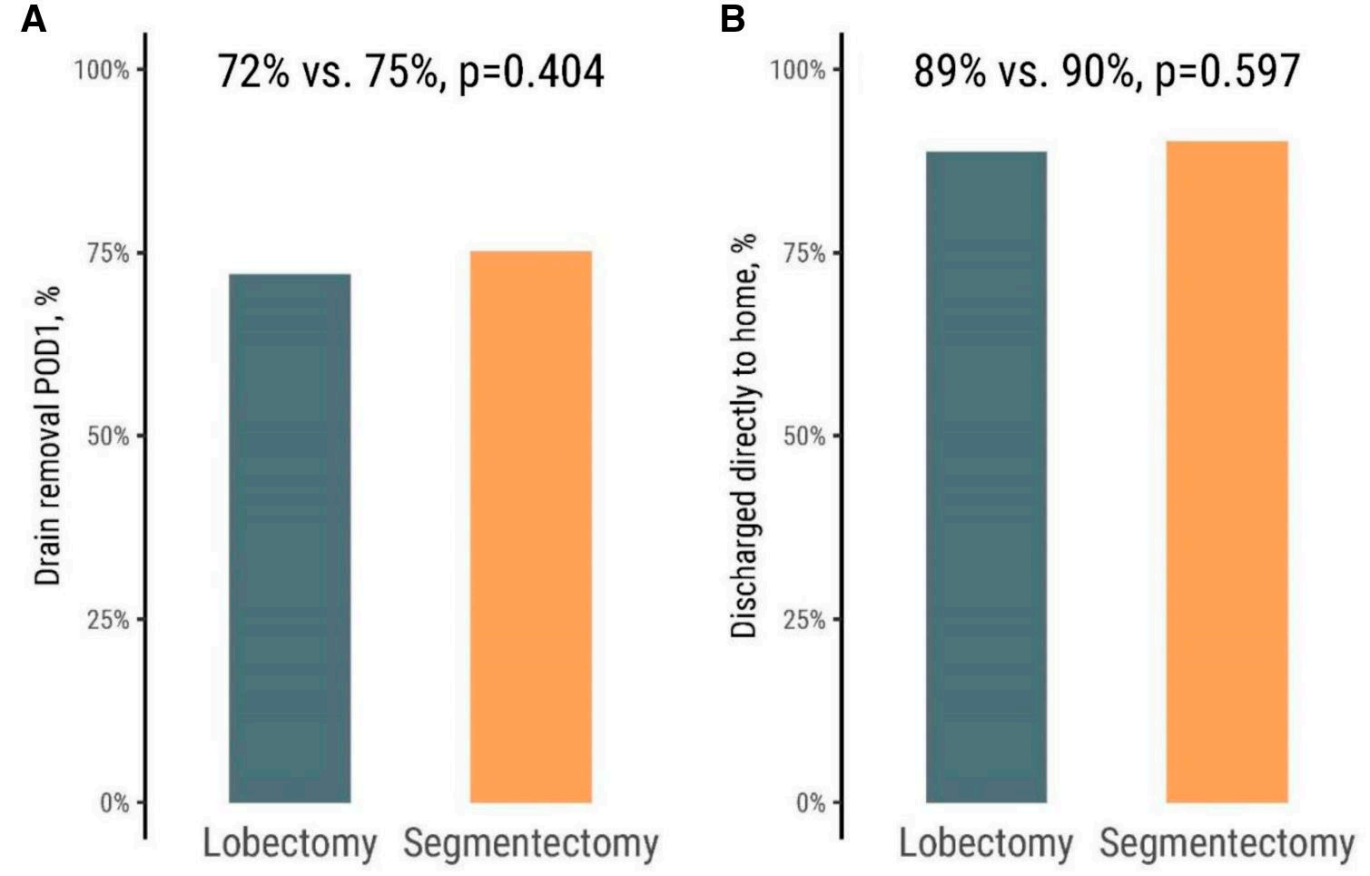
The proportion of patients who underwent uniportal lobectomy or segmentectomy and had their drains removed on postoperative day 1 (**A**) and the proportion who were discharged directly to home (**B**). Analyses were performed for the weighted population. POD: postoperative day

Figure 3 shows the length of the hospital stay for patients who underwent lobectomy and segmentectomy. The median time in hospital was 2 days in both groups, and most patients were discharged on postoperative day 1 or 2. The median chest drainage time was 1 day in both groups. In the lobectomy versus segmentectomy groups, respectively, 72% vs 75% of the patients had their drain removed on postoperative day 1 (*P* = 0.404), and 89% vs 90% were discharged directly home following surgery (*P* = 0.597) (Fig. 4).

## DISCUSSION

In this single-centre analysis of all uVATS segmentectomies and uVATS lobectomies performed between 2017 and 2022, we found no differences in the early postoperative clinical results between the groups. This adds to the growing body of evidence suggesting that, for patients with suitable lesions, uVATS segmentectomy can be implemented as a lung-sparing alternative to uVATS lobectomy without increasing the risk of complications while still maintaining minimal invasiveness.

The implementation of uVATS segmentectomy in our hospital was gradual over the study period. The ratio between segmentectomies and lobectomies decreased over time as mastery of the technique was achieved and the procedure was incorporated into the standard treatment arsenal. Conversion to mVATS or to an open approach occurred evenly during the study period. It could be argued that the threshold for converting to a more familiar approach ought to be low in the implementation of a novel procedure, motivating a high rate of conversion. All but one of the conversions (uVATS lobectomy to thoracotomy) were performed under controlled circumstances, and most were owing to technical difficulties in safely dissecting the anatomy or positioning the endostaplers. The decreasing ratio of lobectomy to segmentectomy seen in conjunction with the low rate of conversions and complications over the study period suggests that the implementation of uVATS segmentectomy could be performed without increased risks for the patients.

There were some differences in preoperative characteristics between the lobectomy and segmentectomy groups in our study. Compared with the lobectomy group, the segmentectomy group was slightly younger and less likely to have a high performance status score, a trend also seen in 1 large study [3] but not in another [22]. The reason for this difference in the present study is unclear. Additionally, more patients who underwent segmentectomy versus lobectomy had undergone previous thoracic surgery. These differences likely reflected the fact that benign disease and metastases were more common in the segmentectomy versus lobectomy groups. However, the differences in age, performance status and previous surgery were adjusted for in the final analysis and found not to have a clinically significant effect on short-term outcomes. Patients who had undergone segmentectomy versus lobectomy were less likely to have undergone preoperative positron emission tomography. However, positron emission tomography is not considered mandatory in the preoperative work-up of pure ground-glass opacity lesions or in benign and metastatic disease at our centre. In cases of repeat thoracic surgery, it is reasonable to assume that a sublobar resection would have been chosen to conserve lung volume. This could account for some of the differences in the frequency of previous thoracic surgery between the segmentectomy and lobectomy groups, reflecting the difference in the proportion of metastatic disease. No significant differences were found in the extent of comorbidities or poor lung function as assessed by forced expiratory volume in 1 s. Moreover, analysis of postoperative events and complications was performed using the weighted samples, for which the aforementioned differences had been adjusted.

In a comparison of postoperative results between VATS segmentectomy and lobectomy by Bédat *et al.* in 2019, the segmentectomy group had shorter drain time and hospital length of stay compared with the lobectomy group [22]. In contrast, neither the drain time nor the length of stay varied significantly between the groups in our study, similar to results in previous studies [8, 9, 11, 23–27]. The lack of difference in drain time and length of stay in the present study further suggests that the more complex dissection of bronchovascular structures and division of lung parenchyma in segmentectomy is not associated with a higher incidence of postoperative air leak. When patients have ongoing air leak, there are multiple strategies at hand. Often, the air leak stops spontaneously as the lung heals over several days; however, this tends to lead to a longer hospital stay. Another strategy is early reoperation to repair the air leakage site. The short average drain time and hospital length of stay in the present study may reflect a greater tendency to adopt the latter strategy as the rate of reoperation was higher compared with results presented by Bédat *et al.* [22]. The hospital stay for the present cohort was short. Since 2017, there has been an early recovery after thoracic surgery regimen in place at our institution with the goal of optimizing pre-, peri- and postoperative care to shorten the hospital stay. This likely impacted the length of stay in the present study. Compared with results from our previous study [13], both drain time and length of stay have shortened over time. In the end, local routines and the organization of healthcare systems have a large impact on data on hospital length of stay, which leads to uncertainty when making comparisons between studies from different centres and countries.

Perioperative lymph node sampling is important for pathological staging of malignant disease. In patients with lung cancer in this cohort, there were fewer lymph node stations sampled in the segmentectomy versus lobectomy groups. The reason for this is not readily elucidated by the data and whether it will impact long-term survival in this cohort is unclear. Speculatively, 1 reason for this difference between the groups could be the surgeon’s decision to avoid extensive lymph node sampling to limit total operative trauma in cases where sampling was not strictly indicated, for instance in patients with ground-glass opacities, owing to patient frailty or in repeat surgery where station 7 had already been dissected. Dissection and removal of all mediastinal lymph nodes is not standard procedure at our institution. The tendency not to sample lymph node stations during minimally invasive segmentectomy, as opposed to lobectomy, was reported previously in 1 study [26] but not in others [9, 27]. The number of resected lymph nodes, but not the extent of resection (segmentectomy/lobectomy) is associated with nodal upstaging from stage 1 disease [28]. This further underlines the need for thorough lymph node sampling in malignant disease regardless of the extent of pulmonary resection so that mediastinal nodal engagement can be discovered on postoperative pathology and timely adjuvant therapy can be started. Guidelines regarding lymph node dissection in segmentectomy specifically are likely to be updated in the near future [5].

The rate of complications was low in both groups in this study. Although estimated blood loss is not reported in the ThoR registry, there was a higher incidence of blood transfusion in the segmentectomy versuss lobectomy groups, a finding not seen in a previous study that was similar to ours [27]. The reason for the higher rate of transfusion is not readily elucidated by the available data. The higher rate could be secondary to differences in perioperative blood loss but could also be owing to preoperative patient characteristics in the segmentectomy groups not reported in data from the ThoR registry. [27] Overall, the rate of complications following segmentectomy in this study was low compared with other studies [8, 9, 11, 22–27]. Discrepancies in complication rates between studies may be owing to varying definitions of what constitutes a complication as well as discrepancies in the likelihood of complications being registered and reported. Karolinska University Hospital is the sole provider of thoracic surgery for the 2.5 million people living in the greater Stockholm area. Patients and all healthcare providers are instructed to refer patients back to our hospital within the first 30 days after surgery for all conditions related to surgery that may require readmission. Additionally, all patients are scheduled for a follow-up visit 4–8 weeks after surgery. Because of this, we feel confident that significant complications were reported. However, data on minor complications, such as superficial wound infections or uncomplicated pneumonia that do not require hospital readmission and are treated in primary care, were not available in the present study. This could be a possible explanation for the difference in complication rates between our study and other studies.

One of the major appeals of segmentectomy over lobectomy is the preservation of lung volume. However, whether this translates to better preservation of lung function is unclear, although a few studies have specifically evaluated differences in measures of lung function. In a meta-analysis, Charloux and Quoix found that 12 months after surgery, segmentectomy resulted in a smaller loss of forced expiratory volume in 1 s compared with lobectomy, but the difference was only 3–13% [29]. In a recent randomized study by Saji *et al.*, the difference was 3.5% and favoured segmentectomy; however, the result was not considered clinically significant [3]. In another randomized study by Altorki *et al.*, the difference at 6 months was 2% [4]. Notably, this study included wedge resection in the sublobar group. Moreover, there is reason to believe that postoperative loss of lung function decreases over time as the lung adapts [3, 29]. A retrospective study evaluating the number of resected segments and loss of lung function found that segmentectomy offered a benefit over lobectomy when only 1–3 segments were removed [30]. Exercise tests, such as the 6-min walk test or ergospirometry, are needed to better categorize differences in loss of lung function following lung resection. Going forward, it would be of interest to see this aspect studied in a randomized trial. Both postoperative measures of lung function and exercise tests were outside the scope of the present study.

### Strengths and limitations

For all patients who underwent uVATS segmentectomy, complete records were available. We feel the study design and available data are robust, and that this study shows how uVATS segmentectomy can be implemented in a “real world” setting where the usual alternative would be lobectomy.

Our study has several important limitations. We used robust statistical methods to address confounding, but residual confounding may still exist. Furthermore, all surgeries were performed at a single centre, and generalizability of these results is thereby limited. Most uVATS segmentectomies at the beginning of the study were performed by 1 surgeon; however, over the study period, all 4 senior surgeons at our centre performed operations in both the segmentectomy and lobectomy groups. Moreover, owing to the selection of suitable patients for segmentectomy, it is expected that patients in the early period were less complex compared to later in the study period. Our study is likely underpowered to detect differences in rare outcomes between the groups, e.g. 30-day mortality. The findings of our study must be interpreted with these limitations in mind. We did not have access to postoperative measures of lung function to assess changes following surgery and whether there were differences between the groups. Finally, although microscopic radicality was assessed and found to be equal in both groups, long-term oncological effectiveness of uVATS segmentectomy must be assessed under different study conditions as the follow-up duration in this study was short. This study primarily aimed to assess the safety of uVATS segmentectomy, not oncological outcomes compared with lobectomy.

## CONCLUSION

We found that uVATS segmentectomy, when implemented gradually, was performed with similar early postoperative clinical results compared with uVATS lobectomy in patients with benign, metastatic or early-stage lung cancer, and that it could be implemented as a standard procedure in the surgical treatment of pulmonary disease.

## Supplementary Material

ezae127_Supplementary_Data

## Data Availability

The data underlying this article are available in the article and in its online supplementary material.

## ABBREVIATIONS

mVATS: Multiportal video-assisted thoracic surgery
RECORD: REporting of studies Conducted using Observational Routinely-collected health Data
STROBE: Strengthening the Reporting of Observational Studies in Epidemiology
ThoR: Swedish national quality register for general thoracic surgery
uVATS: Uniportal video-assisted thoracic surgery
VATS: Video-assisted thoracic surgery
