# Supplementary material for: Uniportal video-assisted thoracic surgery: segmentectomy versus lobectomy—early outcomes
Source: Eur J Cardiothorac Surg. 2024 Mar 28;65(4):ezae127. doi: 10.1093/ejcts/ezae127 (PMC11009028; doi:10.1093/ejcts/ezae127)

Supplementary Material

**Uniportal video-assisted thoracic surgery segmentectomy vs lobectomy – early outcomes**

Erik Sachs^1, 2^, MD, Veronica Jackson^1^, MD, PhD, Mamdoh Al-Ameri^1, 2^, MD, PhD, Ulrik Sartipy^1, 2^, MD, PhD

^1^Department of Molecular Medicine and Surgery, Karolinska Institutet, Stockholm, Sweden

^2^Department of Cardiothoracic Surgery, Karolinska University Hospital, Stockholm, Sweden

| **Supplementary Table 1.** Overview of which segments that were resected by uniportal video assisted thoracic surgery. | |
| --- | --- |
| Segment number | Number of patients (%) |
| 6* | 64 (27.6) |
| 2 | 38 (16.4) |
| 1, and 2 | 34 (14.7) |
| 1 | 20 (8.62) |
| 3 | 13 (5.60) |
| 8 | 13 (5.60) |
| 4 and 5 (left)* | 12 (5.17) |
| 1, 2, and 3 (left)* | 8 (3.45) |
| 8, 9, and 10 | 7 (3.02) |
| 7 and 8 | 6 (2.59) |
| 8, 9, and 10 | 4 (1.72) |
| 10 | 3 (1.29) |
| 3, 4, and 5 | 2 (0.86) |
| 8 and 9 | 2 (0.86) |
| 1 and 3 | 1 (0.43) |
| 2 and 6 | 1 (0.43) |
| 3 and 6 | 1 (0.43) |
| 4 and 6 | 1 (0.43) |
| 4 | 1 (0.43) |
| 7 | 1 (0.43) |
| *These were considered simple segmentectomies, all others were considered complex. | |

| **Supplementary Table 2.** Definition of postoperative events and complications. | |
| --- | --- |
| Outcome | Definition |
| Complications |  |
| No complication | No recorded complication: yes/no |
| Pneumothorax requiring a new drain | Reinsertion of a drain on the operated side: yes/no |
| Arrhythmia | Postoperative arrhythmia requiring treatment: yes/no |
| Stroke/TIA | Postoperative new loss of central neurologic function: yes/no |
| Myocardial infarction | Elevated cardiac biomarkers and at least one of the following: central chest pain, ischemic changes on ECG, new regionality on echocardiogram: yes/no |
| Wound infection | If 2 out of 3 criteria are met; surgical revision, positive culture, treatment with antibiotics: yes/no |
| Pneumonia | If 3 out of 5 criteria are met; temp >38°C, leukocytosis, positive chest X-ray, positive sputum culture, treatment with antibiotics: yes/no |
| Empyema | Temp >38°C or elevated inflammatory biomarkers and purulent pleural drainage: yes/no |
| Lymph leak | Lymphatic pleural drainage: yes/no |
| Recurrent nerve paralysis | Confirmed paresis or paralysis of the vocal cord on the operated side: yes/no |
| Phrenic nerve paralysis | Postoperative paresis or paralysis of the diaphragm on the operated side: yes/no |
| Pulmonary embolism | Positive CT pulmonary angiogram: yes/no |
| Other complication | Any other complication requiring medical care or intervention: yes/no |
| Reoperation | Reoperation for any cause: yes/no |
| Transfusion of blood products | Transfusion of any type of blood product (red blood cells, plasma or platelets): yes/no |
| Station 7 sampled | Station 7 sampled: yes/no |
| ≥3 N2-stations sampled, including station 7 | ≥3 N2-stations sampled, including station 7: yes/no |
| Incomplete resection (on microscopy) | Tumor cells up to the resection margin on microscopy: yes/no |
| Death within 30 days | All-cause mortality within 30 days of surgery: yes/no |
| Death within 90 days | All-cause mortality within 90 days of surgery: yes/no |
| C, Celsius; CT, computed tomography; ECG, electrocardiogram | |

**Supplementary Figure 1.**

Patient flowchart

601 uVATS lobectomies

232 uVATS segmentectomies

81 were excluded (segmentectomies)

37 (2) converted to mVATS

44 (4) converted to open thoracotomy

Included in the analysis

914 planned uVATS lobectomies or segmentectomies

**Supplementary Figure 2.**

Absolute standardized differences before (hollow circles) and after (filled circles) inverse probability of treatment weighting.


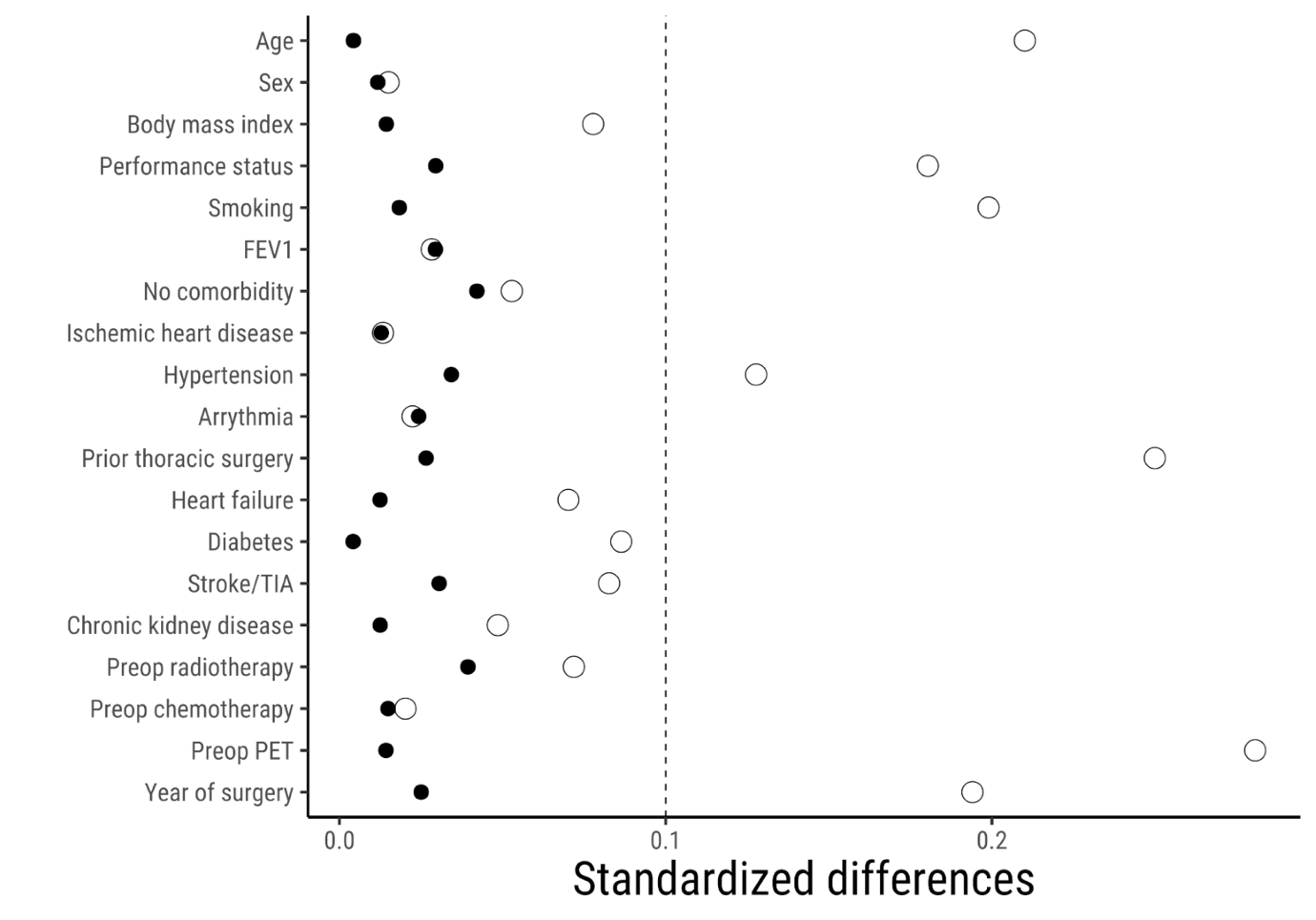

Supplement: ezae127_Supplementary_Data [file ezae127_supplementary_data.zip › Supplemental_Material-revision1_002.docx]
